# The cumulative effect of chronic stress and depressive symptoms affects heart rate in a working population

**DOI:** 10.3389/fpsyt.2022.1022298

**Published:** 2022-10-13

**Authors:** Erika Lutin, Carmen Schiweck, Jan Cornelis, Walter De Raedt, Andreas Reif, Elske Vrieze, Stephan Claes, Chris Van Hoof

**Affiliations:** ^1^Electrical Engineering-ESAT, KU Leuven, Leuven, Belgium; ^2^Imec, Leuven, Belgium; ^3^Department of Psychiatry, Psychosomatics and Psychotherapy, Goethe University, Frankfurt am Main, Germany; ^4^Department of Neurosciences, Psychiatry Research Group, KU Leuven, Leuven, Belgium; ^5^University Psychiatric Centre KU Leuven, Leuven, Belgium; ^6^OnePlanet Research Center, Wageningen, Netherlands

**Keywords:** heart rate, depressive symptoms, chronic stress, circadian rhythm, stress reactivity

## Abstract

**Background:**

Chronic stress and depressive symptoms have both been linked to increased heart rate (HR) and reduced HR variability. However, up to date, it is not clear whether chronic stress, the mechanisms intrinsic to depression or a combination of both cause these alterations. Subclinical cases may help to answer these questions. In a healthy working population, we aimed to investigate whether the effect of chronic stress on HR circadian rhythm depends on the presence of depressive symptoms and whether chronic stress and depressive symptoms have differential effects on HR reactivity to an acute stressor.

**Methods:**

1,002 individuals of the SWEET study completed baseline questionnaires, including psychological information, and 5 days of electrocardiogram (ECG) measurements. Complete datasets were available for 516 individuals. In addition, a subset (*n* = 194) of these participants completed a stress task on a mobile device. Participants were grouped according to their scores for the Depression Anxiety Stress Scale (DASS) and Perceived Stress Scale (PSS). We explored the resulting groups for differences in HR circadian rhythm and stress reactivity using linear mixed effect models. Additionally, we explored the effect of stress and depressive symptoms on night-time HR variability [root mean square of successive differences (RMSSD)].

**Results:**

High and extreme stress alone did not alter HR circadian rhythm, apart from a limited increase in basal HR. Yet, if depressive symptoms were present, extreme chronic stress levels did lead to a blunted circadian rhythm and a lower basal HR. Furthermore, blunted stress reactivity was associated with depressive symptoms, but not chronic stress. Night-time RMSSD data was not influenced by chronic stress, depressive symptoms or their interaction.

**Conclusion:**

The combination of stress and depressive symptoms, but not chronic stress by itself leads to a blunted HR circadian rhythm. Furthermore, blunted HR reactivity is associated with depressive symptoms and not chronic stress.

## Introduction

Depression is associated with pronounced autonomic nervous system abnormalities. Patients with Major Depressive Disorder (MDD) show higher baseline levels of heart rate (HR) and altered heart rate variability (HRV) [e.g., lower Standard Deviation of Normal-to-Normal Intervals (SDNN), Root Mean Square of Successive Differences (RMSSD), low-frequency and high frequency (HF) HRV] (1–3). These baseline differences are paired with blunted reactivity during exposure to acute and repeated psychological stress (2–5). Furthermore, differences of HR and HRV at baseline extend to abnormalities of HR circadian rhythm in severe depression (6, 7). A circadian rhythm is an oscillation of a process over 24 h. HR, for example, rises strongly in the morning, reaches a peak before noon, decreases again and reaches another peak in the afternoon, after which it keeps on lowering during the night until the next morning (8). Abnormalities within this rhythm may be used to discriminate between cases and controls (4, 9). Indeed, it is well known that patients with MDD show altered circadian rhythms and diurnal mood variation (10). Analyzing HR/HRV, particularly during the night period may provide more insight into potential pathological mechanisms.

Interestingly, the association of depressive symptoms and HR/HF-HRV is not as clear-cut. Depressive Recently, Jarczok and colleagues showed that depressive symptoms were associated with reduced 24-h cardiac vagal variation in men, but increased variation in women (11). Interestingly, in their large-scale longitudinal study, Jandackova et al. (12) did not find a cross-sectional association between HR/HRV measures and depressive symptoms at baseline. However, in a predictive analysis, the authors showed that lower HR and higher HF-HRV predicted a lower likelihood of depressive symptoms at follow-up, 10 years later. Inversely, the presence of depressive symptoms at baseline was not associated with HR/HRV measures at follow-up (12). Similar findings have been reported by others: It seems that particularly higher levels of HRV are indicative of a better outcome to treatment [e.g., (13)]. It should also be noted, that resting state HR/HRV alterations do occur in various other psychiatric [e.g., post-traumatic stress disorder (14), anxiety disorders (15)] and somatic [e.g., type 2 diabetes (16), fibromyalgia (17)] disorders and should not be understood as a diagnostic biomarker, but rather as general risk assessment that if combined with other measures, could be helpful in identifying at-risk patients.

While resting state physiological data are informative, functional data provides important information on the capacity to adapt to environmental demands. Stress reactivity studies in healthy people with depressive symptoms are ambiguous: increased (HR) reactivity (18), decreased reactivity (19), or no changed reactivity to a mental stressor (20, 21) have been reported.

A big caveat of the above studies, and possibly a reason for the heterogeneous findings, is that they did not account for the effects of chronic stress. Amongst other factors which can influence HR and HRV readouts, such as age and sex (22), poor cognitive function (23, 24), mental fatigue (25), metabolic syndrome (26), temperature (27), and possibly, other lifestyle factors [e.g., physical activity, alcohol use, smoking (22)], chronic stress may increase HR reactivity and/or decrease HRV (28, 29), although some reports did not find an effect (22). Chronic stress is also a well-known risk factor for psychopathology: the experience of chronic stress (i.e., the real or perceived threat to an individual’s psychological or physiological integrity) (30, 31) has been linked to both the development (32) and the maintenance (33) of several mental disorders, one of which is MDD. In contrast to the blunted stress reactivity observed in MDD, high perceived stress and/or negative life events may increase HR reactivity to stress (34). Changes in HR/HRV may thus represent the consequence of chronic stress, which can precede the development of depressive symptoms [e.g., (12, 29)]. Depression and/or depressive symptoms are often associated with the subjective experience of chronic stress (35); the direction of causality, i.e., whether chronic stress precedes depression or whether depression leads to high subjective stress, is unclear and may vary between individuals.

As initially proposed by McEwen, the identification of subclinical cases before emergence of psychopathology may be key to answer these questions (30). Therefore, we set out to investigate the effects of chronic stress on circadian rhythms and stress reactivity in the Stress in the Work Environment (SWEET) study, which studies a large sample of individuals from the healthy working population. The participants were specifically selected to have (a) no chronic stress and no depressive symptoms, (b) chronic stress but no depressive symptoms, (c) depressive symptoms but no chronic stress, and finally (d) depressive symptoms and high chronic stress.

We aim to investigate whether the effect of chronic stress on HR circadian rhythm is (a) dose dependent and (b) different in participants with, compared to participants without depressive symptoms. We hypothesize that chronic stress and depressive symptoms gradually increase basal HR levels, and that the combination of both, high chronic stress and depressive symptoms results in a biological profile similar to patients with depression. Secondly, we investigated if chronic stress and depressive symptoms alter HR reactivity to an acute stressor. We hypothesize that high depressive symptoms are associated with blunted stress reactivity, while high stress is associated with increased stress reactivity.

## Materials and methods

### Participants

This study is part of the Stress in the Work Environment study (SWEET-study; *n* = 1,002) described in Smets et al. (36), which was approved by the Ethics Committee Research UZ/KU Leuven (approval number: S57916). Participants were recruited in 11 technology-oriented, banking, and public sector companies. Participants with heart disease or any psychiatric disorder based on self-report were excluded from analysis. Participants who did not report a psychiatric disease but were currently taking psychotropic medication were also excluded. Cholesterol-lowering supplements or medication was permitted if no heart disease was specified. Participants did not receive any reward for participating in the study apart from having a chance at winning a restaurant or travel voucher.

### Procedures

#### Questionnaires

Before the experiment, participants completed an intake questionnaire which gathered personal information, such as age, sex, health problems, work situation, and lifestyle, and four psychological questionnaires: The Perceived Stress Scale with 10-items (PSS-10), the Pittsburgh Sleep Quality Index (PSQI), and the Depression Anxiety Stress Scale with 21 items (DASS-21).

#### Ambulatory monitoring

On Thursday morning, participants received a chest patch [hardware system of (37)] which received regulatory approval and measures the electrocardiogram (ECG) and tri-axis acceleration (Acc) at a sampling rate of 256 and 32 Hz, respectively. Though higher sampling rates are preferred in HRV research, a sampling rate of 256 Hz has been reported as sufficient (38) and was preferred in this large-scale ambulatory study to limit memory and storage issues. Participants were asked to wear the chest patch day and night for a total of 5 days (Thursday–Monday morning) and to remove it only during vigorous physical activities. For more details on the data collection, see Smets et al. (36).

#### Stress task

On the first day (Thursday) of the experiment, participants were asked to complete a modified version of the Montreal Imaging Stress Task (MIST) (39) to induce moderate stress. The MIST (hereafter referred to as stress task) consists of a resting, a training, and an experimental condition. In this study, participants completed the task on a mobile app: a 5-min rest period (relaxing music and images), a 5-min training period, a 5-min stress task and a 5-min recovery period. For details on the task, see Supplementary Information 1.

#### Data pre-processing

The mean HR(V) and activity levels were calculated in windows of 5 min with 4 min of overlap, analog to Schiweck et al. (4) and Smets et al. (36), following the minimum window length required to calculate HRV features. The activity level of the participants was calculated as the standard deviation of the magnitude of acceleration (Std Acc) as derived from the accelerometer of the ECG patch. As ambulant physiological recordings may be corrupted by motion artifacts and poor sensor attachment, the acceptability of the recorded ECG was assessed using a quality indicator (QI) derived from Orphanidou et al. (40) and previously implemented by Smets et al. (36). For details see Supplementary Information 2. The HR(V) data was filtered using the QI. All 5 min windows with an average QI below 0.8 were excluded. In addition, all segments of high activity (Std Acc > 0.04) were excluded to obtain a clear result without the confounding effect of physical activity. Finally, all retained windows were averaged into hourly measurements.

The ECG data recorded during the stress task was divided into windows of 1 min, without any overlap, from which the mean HR was calculated. Windows with an average QI below 0.8 were excluded. The retained windows were averaged per condition of the stress task.

### Statistical analysis

All statistical analysis was performed in *R version 3.6.1* (41). Graphical representations were performed with *Python version 3.7.* Relevant variables were age, biological sex, hours of physical activity per week, smoking behavior, DASS scores, and PSS scores. For simple group comparisons, the Wilcoxon rank sum test was performed. For multiple group comparisons, the Kruskal-Wallis test was used. If significant, it was followed by Dunn’s test with a Benjamini-Hochberg correction. Missing weight or length data was imputed by the median of the included population. Hours of physical activity per week was enquired as the total hours of sports performed per week with 5 suggested categories: 0, 0–1h, 1–3h, 3–5h, and > 5 h. These values were transformed into a numeric variable ranging from 0 to 4. For circadian variation, recordings were required to include an average HR for at least every hour of a 24 h day (i.e., a minimum of 24 datapoints without constraints for within-day consecutiveness), in which the hourly average was derived from at least 5 data windows. If this requirement was violated, the corresponding participant was excluded. For stress reactivity analyses during the stress task, only participants who completed the MIST and for whom physiological recordings were of sufficient quality were included.

#### Definition of depression and stress subgroups

To define our population of interest, with and without symptoms of depression and low or high stress, respectively, we used the DASSD [Cronbach’s alpha: 0.81 (42)] and PSS scores [Cronbach’s alpha: 0.78 (43)]. First exploratory dimensional analyses, using HR averages per day and night, indicated the possible importance of second order terms in the PSS and/or DASSD (see Supplementary Table 2).

Since several model terms were already used to model circadian rhythm, we decided against further dimensional analyses, as these would require the inclusion of second order terms for both the PSS and DASSD in interaction with all the harmonic terms. To preserve interpretability and allow for non-linear relationships, we opted for group-based analyses.

The DASSD was used to define groups experiencing no or only low depression scores, and those with high levels, following the official cut-off score of 6 as recommended by Lovibond and Lovibond (44). We opted for a binary distribution due to the expected, non-normal distribution of depression scores in a working population which was confirmed by a skewness of 1.67 and kurtosis of 6.33 within the DASSD (skewness > 1, the distribution is heavily skewed; kurtosis > 3, the distribution has heavier tails than a normal distribution). Regarding the PSS, several cut-offs are mentioned in the literature, but since the PSS is not a diagnostic instrument, no validated cut-offs are given. Given our interest in the difference between low/normal, medium/high, and extreme stress (often present in depression), participants were grouped into three categories using our own, population-derived cut-off values: (1) normal stress levels (lower or equal to the 75th percentile of the PSS scores), (2) high stress levels (between the 75th and the 95th percentile), and (3) very high stress (above the 95th percentile). This corresponded to a PSS value of < 18 for normal stress, between 18 and < 24 for high stress or > 24 for extreme stress. These values are congruent with the values published by Cohen and Janicki-Deverts (45) in their large survey of the normal population using the PSS-10: Mean PSS scores in 2009 were situated around 17 (16.38–17.46) with a standard deviation of around 7 (7.07–7.83) for the population aged 25–54, which is comparable to our sample.

#### Circadian rhythm

##### Model development for heart rate

We performed a group-based time-series analysis in which we studied HR, for every group, at consistent time intervals, i.e., 1 h, while modeling time dependencies between the intervals. For this, we used a linear mixed model, predicting HR with the independent variables age, sex, Body Mass Index (BMI), smoking, depressive symptom group, and chronic stress group. The hourly average activity level (Std. Acc), hereafter referred to as the activity index, was also included as a covariate to control for HR recovery following (the excluded) periods of high physical activity. All numerical variables were rescaled to range from 0 to 1. As HR shows diurnal fluctuations (8, 46), we introduced harmonic terms with periods of 24, 12, 8, and 6 h as described in the literature (47, 48). Via interactions with the factor groups, these terms allow for the estimation of group-related diurnal differences. A random intercept was included per subject, as well as random slopes for all harmonic terms. The latter allow us to model person-to-person variability. Due to slight deviations from model assumptions, HR data was log transformed. As the effect of sleep quality was not of primary interest, its relevance was only tested in the final model after the model selection procedure. In a second step, separate models were built for healthy participants with high and low levels of depressive symptomatology to investigate whether differential effects were present.

##### Model development for heart rate variability

Regarding HR variability, we aimed at studying vagal modulation, as this has been linked with stress vulnerability and reactivity (49, 50) and depression (51). Cardiac vagal tone can be approximated through the HF variations of the RR interval, which primarily reflects the activity of the parasympathetic nervous system (52). The root mean squared successive differences (RMSSD) is a time domain measure that represents short-term variation, and therefore correlates with the power in the HF band. For long-term, ambulatory ECG recordings, it has been recommended to look into RMSSD rather than the HF-HRV (53). Since we were not able to control for respiratory measures and RMSSD is known to be less influenced by respiration than HF-HRV (54), we chose RMSSD as outcome.

Pre-processing revealed that the day-to-day variability within RMSSD was substantially higher than within HR (see Supplementary Table 1 for the comparison). This might have been a consequence of the ambulatory set-up, in which the ECG recording is subject to artifacts and noises: baseline wandering, electromyogram interference, powerline interference, and motion artifacts. As HRV measures require high temporal accuracy for R-wave peak identification, part of the variability may have originated from poor signal quality. Consequently, the circadian model, as explained in the previous section, did not reach good model fit. To reduce the influence of motion, we limited the analysis of RMSSD to night-time analyses (from 10 p.m. to 6 a.m.).

Similar to the HR analysis, we performed a group-based time-series analysis using a linear mixed effects model. In this model, we predicted the hourly average for RMSSD with the independent variables age, sex, BMI, smoking, activity index, depressive symptom group, and chronic stress group. In contrast to the model for HR, we added time directly to the model, without harmonic terms. Interactions between the time variable and the factor groups allowed for the estimation of group-related differences in night-time recovery, i.e., the night-time slope of RMSSD. The variable time ranged from 1 to 8, representing the hours between 10 p.m. and 6 a.m. A random intercept was included per subject, as well as a random slope for time. Due to slight deviations from model assumptions, RMSSD data was log transformed.

##### Model selection

For HR, a final model was selected using backward stepwise elimination based on the Akaike information criterion (AIC). The AIC is an estimator of prediction error which promotes models with a high goodness-of-fit and penalizes overly complex ones (55). The stepwise elimination process was performed using the R-package “buildmer” with the parameter “crit” set on “AIC” and the parameter “direction” set on the default combination (“order, backward”) to first make sure that the model converges and to then perform backward elimination (56). The retained variables were finally tested in the R-package “lme4.” Importantly, in contrast to the traditional cut-off based on a *p*-value of 0.05, the AIC-based model selection allows for the inclusion of variables with a *p*-value above the significance level of 0.05 if these variables improve the goodness-of-fit of the model significantly. With regard of RMSSD, we did not perform a stepwise elimination but estimated the full model.

#### Stress reactivity

In a linear mixed model, we predicted HR with the independent variables: depressive symptom group, chronic stress group and stress exposure. A random intercept was included per subject. To investigate the effect of depressive symptoms and chronic stress on stress reactivity, both group factors were added in interaction with the stress exposure. No stepwise elimination was performed. Per grouping, the conditions were compared in a pairwise manner while using a Benjamini-Hochberg correction. We did not analyze reactivity in RMSSD for the reasons explained in section “Model development for HR variability.”

## Results

### Sample characteristics

A total of 1,002 participants took part in the SWEET study. Among these participants, 104 did not have any (good quality) ECG data and 181 did not have enough data, i.e., at least one data point for every hour in a day, to perform circadian modeling. This data loss found its origin in several issues inherent to ambulatory data collection such as participant drop-out within 24 h, poor signal quality because of bad sensor adhesion or vigorous motion and sensor failure because of water damage (e.g., while showering). An additional 153 participants did not fill in the questionnaires and 48 participants were removed from the data set based on the exclusion criteria [*n* = 13 psychiatric disorders (anxiety, ADHD, depression, or other)], *n* = 2 intake of psychotropic medication without diagnosis, *n* = 17 heart diseases with medication intake such as beta-blockers, *n* = 15 heart diseases without medication intake, *n* = 1 chronic disease (neuropathy), resulting in a total of 516 participants included for analyses [mean age = 39.44 (SD 10.21), mean BMI = 24.20 (SD 3.78), number of women = 243 (47%), mean PSS = 14.21 (SD 6.01), mean DASSD = 2.50 (SD 2.91), mean HR = 67.87 BPM (SD 7.53)]. Participants were mostly highly educated (34.9% graduate school, 7.3% secondary school, 57.8% university). On average, participants had 83.8 (SD 17.7) hourly averages of valid physiological recording spread over the entire period of approximately 104 h (Thursday morning 9 a.m. to Monday evening 5 p.m.). The group of participants who were excluded because of insufficient ECG data after pre-processing (*n* = 285) did not differ significantly in age, sex, smoking behavior, education, PSS, and DASSD from the final group. However, the final group reported higher levels of physical activity than the excluded group (*p*-value = 0.035).

#### Stress subgroups

Three hundred ninety participants (75.59%) had low to normative chronic stress levels (PSS ≤18) (hereafter: normative stress group), 106 (20.54%) had moderate to high chronic stress levels (18 < PSS 24) (hereafter: high stress group) and 20 (3.86%) had extreme chronic stress (PSS > 24) (hereafter: extreme stress group). *Post hoc* comparison using Benjamini-Hochberg corrections indicated that the high stress group performed fewer hours of physical activity per week and had a significantly higher HR than the normative stress group (z = 2.564, *p* = 0.031 and z = 2.505, *p* = 0.037, respectively). Fewer women were in the normative stress group compared to the other groups (normative—high; *p* = 0.002 normative—extreme; *p* = 0.011). The normative stress group also indicated better sleep quality than the other groups (normative—high and normative—extreme; *p* < 0.001). All groups differed in their DASSD scores (high—extreme; *p* = 0.015, other; *p* < 0.001). No other variables differed significantly between groups. Sample characteristics and statistical tests for stress subgroups can be found in Supplementary Table 3.

#### Depression subgroups

Four hundred sixty (89.15%) participants scored below the cut-off for depressive symptoms and 56 (10.85%) scored above the cut-off for depressive symptoms. No significant differences were present for age, sex, BMI, smoking behavior, HR, and Std. Acc. Participants high in depressive symptoms performed significantly less sport per week (*W* = 14936, *p* = 0.042), had significantly different PSS scores (*W* = 4502, *p* < 0.001) and significantly poorer sleep quality (*W* = 7204.5, *p* < 0.001). Of the participants with low depressive symptoms, the majority had normative stress levels (*n* = 371, 80.65%), a smaller subset had high (*n* = 79, 17.17%) and few participants had extreme (*n* = 10, 2.17%) stress levels. In contrast, the majority of the participants with high depression scores also had high stress levels (*n* = 27, 48.21%). Nineteen participants within this group (33.93%) had normative stress levels and 10 (17.86%) had extreme stress levels. An overview of the sample characteristics for these combined groups can be found in Table 1, sample characteristics and statistical tests for subgroups separately can be found in Supplementary Table 4.

**TABLE 1:** Sample characteristics.

Depressive symptoms	1 low			2 high					
Chronic stress	A Normal (*n* = 371)	B High (*n* = 79)	C Extreme (*n* = 10)	D Normal (*n* = 19)	E High (*n* = 27)	F Extreme (*n* = 10)			
		
	Median (IQR)	Median (IQR)	Median (IQR)	Median (IQR)	Median (IQR)	Median (IQR)	X^2^-value	*P*-value	Significant differences
Age	39.00 (16.00)	38.00 (17.50)	41.50 (11.00)	45.00 (23.00)	36.00 (16.00)	43.00 (9.50)	3.05	0.693	
BMI	24.09 (4.62)	22.65 (4.88)	22.22 (10.50)	24.00 (1.64)	23.89 (6.81)	24.67 (1.07)	7.18	0.208	
Hours of physical activity	2 (2)	2 (2)	1.50 (1.75)	1 (2)	2 (1.75)	2 (1.75)	12.90	**0.024**	1 ↔ 2^a^
Sex female	42.05%	63.21%	80.00%	36.84%	55.56%	70.00%	20.14	**< 0.001**	A ↔ B
Smoking	7.28%	5.06%	0.00%	10.53%	11.11%	10.00%	2.38	0.795	
PSQI^b^	4 (2.5)	6 (3)	5.5 (3.5)	5 (2.75)	5 (3)	8 (2.75)	49.42	**< 0.001**	A ↔ B,D,E,F
PSS	12 (6)	21 (2)	26.5 (2.75)	15 (3)	23 (3.5)	27.00 (2.75)	296.79	**< 0.001**	1 ↔ 2 A ↔ B,C,D,E,F D ↔ A,B,C,E,F
DASSD	1 (2)	2 (3)	4 (2.5)	8 (2)	9 (2)	9 (3)	194.14	**< 0.001**	12 A ↔ B,C,D,E,F B ↔ D,E,F
HR (in bpm)	66.96 (10.31)	68.12 (9.89)	69.51 (7.22)	66.03 (8.15)	68.92 (8.17)	65.30 (6.34)	8.87	0.114	
RMSSD (in ms)	52.34 (25.09)	54.61 (26.76)	69.11 (38.70)	66.03 (15.54)	53.32 (12.61)	53.61 (17.91)	1.83	0.873	
Std. Acc	0.0411 (0.0123)	0.0409 (0.0123)	0.0369 (0.0112)	0.0337 (0.0130)	0.0389 (0.0111)	0.0443 (0.0150)	5.21	0.391	

Differences are indicated for both depression subgroups and stress subgroups.

BMI, Body Mass Index; PSQI, Pittsburg Sleep Quality Index; PSS, Perceived stress scale; DASSD, Depression scale of the Depression Anxiety Stress scale; HR, average heart rate; RMSSD, root mean square of successive differences; Std. Acc, standard deviation of the magnitude of acceleration.

Bold, *p* < 0.05.

Significant differences between groups as determined by *post hoc* comparisons are indicated by the symbol ↔.

^a^Rejection of Kruskal Wallis followed by no significant differences in corrected Dunn’s test for A,B,C,D,E,F.

^b^The PSQI included missing values. The true number of entries were the following: *n_A_* = 347, *n_B_* = 73, *n_C_* = 8, *n_D_* = 18, *n_E_* = 24, *n_F_* = 10.

#### Stress task

A subset of the participants completed the MIST (*n* = 194). This subset did not differ significantly from the overall sample regarding age, BMI, sex, PSS, and DASSD (*p* > 0.05) Within the subset, 180 participants had low depression scores and 14 high depression scores. Regarding chronic stress, 152 participants had normative stress levels (PSS 18), while 42 participants had high or extreme stress levels (PSS > 18). Given the low number of participants, we decided against further subgrouping of the high stress group. Sample characteristics of respective subgroups can be found in Supplementary Tables 5, 6.

### Circadian rhythm is influenced by chronic stress in healthy participants

**FIGURE 1:**
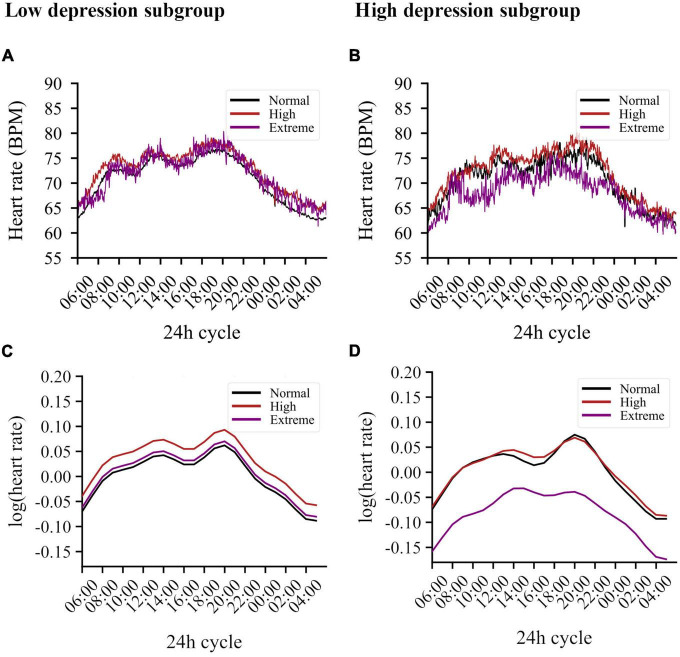
Circadian variations in heart rate averaged per stress subgroup for both low and high depression scores. **(A)** Averaging results for the low depression subgroup. Chronic stress levels are indicated by color: normative levels in black, high levels in red, extreme levels in purple. **(B)** Averaging results for the low depression subgroup. **(C)** Simulation results derived from the model for the low depression subgroup. The graph illustrates the log-transformed HR derived from this model using only the harmonic and stress-related terms, therefore, without the influence of BMI, sex, activity, and smoking behavior. Participants with high levels of chronic stress show the highest increase in HR. **(D)** Simulation results derived from the model for the high depression subgroup. Whereas the negative main effect of the 12 h-based sine wave causes an increase in the morning and evening, the positive interaction of this wave with extreme stress counteracts this, resulting in a diminished evening peak and an elevated plateau in the afternoon. Similarly, the positive interaction including the 8 h-based cosine wave counteracts the negative main effect of this wave, resulting in, among other effects, an additional reduction of the evening peak in participants with extreme stress.

Figures 1A,B present circadian variations in HR for participants with low and high depression scores, respectively. The final model (AIC = −84655.2, LogLik = 42396.6, and R^2^_*conditional*_ = 0.75) retained several significant covariates (see Supplementary Table 7 for a detailed overview of estimates), a main effect for chronic stress, and a three-way interaction for the 8-h harmonic regression term, chronic stress, and depressive symptoms (main effect stress: *F* = 2.849, *p* = 0.059, interaction: *F* = 2.489, *p* = 0.084). Given the significant interaction and contrasts (*b* = 0.018, se = 0.008, *t* = 2.184, *p* = 0.030), we opted for split-group analysis by depression category to further examine the effects of chronic stress.

In the group with low depression scores (*n* = 460), high chronic stress was associated with higher basal HR levels (*b* = 0.031, se = 0.013, *t* = 2.318, *p* = 0.021) when compared to normative stress. HR in the group with extreme stress did not differ from the normative stress group (see Figure 1C). Important covariates in this model were BMI (*p* < 0.001), sex (*p* < 0.001), activity index (*p* < 0.001), and smoking (*p* = 0.073). See Supplementary Table 8 for a detailed overview of estimates.

For the group with high depressive symptoms (*n* = 56), chronic stress also had a significant main effect on the circadian rhythm (*F* = 3.644, *p* = 0.026). However, in contrast to the low depressive symptom group, individuals with extreme stress had *lower* HR (*b* = −0.084, se = 0.035, *t* = −2.408, *p* = 0.016) compared to the normative stress group, while high stress did not change basal HR. In addition, extreme stress altered the circadian rhythm significantly [extreme stress*sin ($\frac{2\,\pi }{12}$h), *b* = 0.022, se = 0.010, *t* = 2.176, *p* = 0.030; and extreme stress*cos ($\frac{2\,\pi }{8}$h), *b* = 0.011, se = 0.005, *t* = 2.136, *p* = 0.033]. These effects resulted in blunted circadian variability of HR in the extreme stress group, noticeable by the elevated plateau in the afternoon and the diminished evening peak. People with high depressive symptoms and high chronic stress show no difference in HR compared to the normative chronic stress group (with high depressive symptoms), see Figure 1D for the simulated regression line. Several covariates were retained: sex (*p* < 0.001), activity index (*p* < 0.001), and smoking (*p* = 0.025). Age and BMI were not retained in the model. See Supplementary Table 9 for a detailed overview of estimates.

When hours of physical activity performed per week was included, the models differed slightly. The altered models are presented in Supplementary Tables 10–12. In the overall model, the *p*-value for the main effect of high chronic stress was reduced to a trend for significance (*p* = 0.081). In the group model for low depressive symptoms, chronic stress was dropped after model selection.

The model for night-time RMSSD reached moderate model fit (AIC = 9796.1, LogLik = −4876.0, *R*^2^ = 0.63), but did not show any significant group effects. Only age, BMI, activity index and the time-point were significant predictors (see Supplementary Table 13). Though insignificant, we explored possible trends for the group effects. RMSSD baseline scores showed a positive effect for stress (high stress: *b* = 0.041, se = 0.047, *t* = 0.863, *p* = 0.389; extreme stress: *b* = 0.177, se = 0.120, *t* = 1.474, 0.141) and a positive effect for depressive symptoms (*b* = 0.088, se = 0.088, *t* = 1.000, *p* = 0.318). However, the interaction of chronic stress groups (both high and extreme) and depressive symptoms had a reducing effect when combined (high stress × dep.: *b* = −0.099, se = 0.122, *t* = −0.814, *p* = 0.416; extreme stress × dep.: *b* = −0.210, se = 0.189, *t* = −1.109, *p* = 0.268). This suggests that the combination of chronic stress and depressive symptoms has a different effect on RMSSD than their mere summation. Regarding the slope in night-time RMSSD, all group effects had a negative or near-zero positive estimate, and general trends were not established.

### Depressive symptoms are associated with lower stress reactivity, chronic stress with higher stress reactivity

For the overall group, reactivity to the stress task (MIST) revealed a significant main effect for the stress task (*F* = 4.289, *p* = 0.005) with increased HR during stress compared to baseline (*b* = 0.028, se = 0.005, *t* = 5.625, *p* < 0.001). While the overall ANOVA showed no significance for the interactions of chronic stress or depression group with stress exposure for the overall group (*F* = 1.868, *p* = 0.134 and *F* = 1.886, *p* = 0.131), the summary output of the model at the factor level showed that chronic stress and depressive symptoms had opposed effects: high chronic stress was associated with slightly higher HR during stress exposure (*b* = 0.025, se = 0.011, *t* = 2.274, *p* = 0.023), while high depressive symptomatology modestly reduced stress reactivity (*b* = −0.037, se = 0.018, *t* = 2.104, *p* = 0.036). See Table 2 for a detailed overview of estimates.

**TABLE 2:** Result of the linear mixed model on reactivity during the stress task (MIST).

	Estimate	Std. error	*T*-value	*P*-value
Intercept	4.239	0.011	391.421	**< 0.001**
Depressive symptoms (high)	0.076	0.011	1.949	0.053
Stress (high)	–0.018	0.039	–0.719	0.473
MIST (training)	0.006	0.005	1.240	0.216
MIST (stress)	0.028	0.005	5.625	**< 0.001**
MIST (recovery)	0.0003	0.005	0.072	0.943
MIST (training)* dep (high)	–0.003	0.018	–0.169	0.866
MIST (stress)* dep (high)	–0.037	0.018	–2.104	**0.036**
MIST (recovery)* dep (high)	–0.020	0.018	–1.114	0.266
MIST (training)* stress (high)	0.013	0.011	1.124	0.261
MIST (stress)* stress (high)	0.025	0.011	2.274	**0.023**
MIST (recovery)* stress (high)	0.007	0.011	0.595	0.552

Results are presented for the prediction of heart rate after log transformation, including a random intercept per participant.

Bold, *p* < 0.05.

**FIGURE 2:**
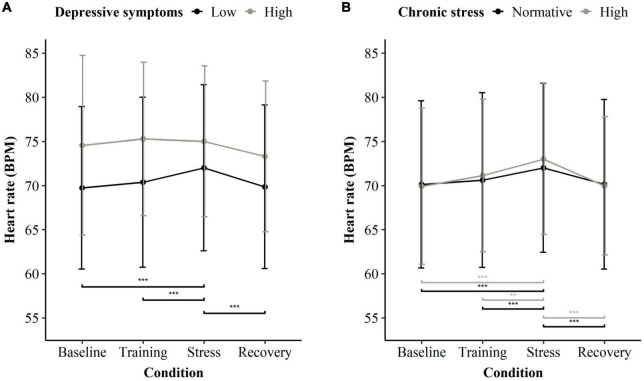
Heart rate averaged per condition of the stress exposure (MIST) and stress/depression subgroup. **(A)** Mean heart rate for participants low in depressive symptoms (black), *n* = 180, and high in depressive symptoms (gray), *n* = 14. All participants, regardless of their chronic stress level, were considered. **(B)** Mean heart rate for participants with normative chronic stress (black), *n* = 152, and high chronic stress (gray), *n* = 42. All participants, regardless of their depressive symptoms, were considered. Significant differences are indicated in a pairwise manner between baseline, stress, recovery, and within subgroups. **p < 0.01 and ***p < 0.001.

Figure 2 shows the pairwise comparison within depressive symptoms groups (A) and within chronic stress groups (B). Participants with low depressive symptoms had significantly higher HR during stress (pairwise contrasts compared to baseline *b* = 0.032, *p* < 0.001; and to the training phase *b* = 0.024, *p* < 0.001) and showed significant recovery after the stressor (pairwise contrast: *b* = −0.031, *p* < 0.001). However, participants with high depressive symptoms showed no significant reactivity or recovery to the stressor for any period (pairwise contrasts all *p* > 0.470). The groups did not differ significantly in HR during the baseline condition (Supplementary Table 4). Estimates per model term are presented in Supplementary Table 14, individual results and characteristics can be found in Supplementary Table 15 and Supplementary Figure 1 regarding the participants with depressive symptomatology. Regarding chronic stress, both participants with normative chronic stress and high chronic stress showed a significantly increased HR during the stress task in comparison to baseline and recovery (Supplementary Table 16).

## Discussion

We here present results from the SWEET study, which allowed us to assess the cumulative effect of stress and depressive symptoms on HR circadian rhythm, night-time RMSSD and HR in response to an acute mental stressor.

We show that chronic stress does not simply lead to an increase in HR but is graded and varies in function of depressive symptoms. Without depressive symptoms, high chronic stress modestly increases basal HR but has no effect on circadian rhythm. In contrast, in those with depressive symptoms, high chronic stress does not change basal HR, nor circadian rhythm; however, the few participants experiencing extreme chronic stress and depressive symptoms showed a lower overall HR paired with an atypical, flattened circadian rhythm variation. In the MIST task, depressive symptoms were associated with blunted HR reactivity while chronic stress with higher HR reactivity. Although this finding must be considered preliminary due to the small group size (*n* = 10), it may have important implications for pathophysiology of depression. In addition, we did not find any significant effects of depressive symptoms and/or stress on RMSSD during the night. Blunted stress reactivity has previously been linked to depression (57), and chronic stress has been hypothesized to be a major factor in the pathogenesis of depression (58). However, given the largely overlapping effect of both, it was previously difficult to show this relationship experimentally. Here, we show that blunted HR reactivity is explained by depressive symptoms and not exposure to high chronic stress when including both in the analysis.

Others have previously shown that 24-h HR is positively correlated with (work-related) stress (29, 59), and have reported associations with various HRV measures (28, 59–61). However, to our knowledge, these studies have mostly used average HR over 24-h intervals and assessed either stress or depressive symptoms, but not both. Few studies specifically assessed circadian rhythm in HR or HRV, some of which assessed chronic stress in shift workers (62, 63), or mood disorders (64), or depressive symptoms (11, 65). In other parameters than HR and HRV, there has been ample research on circadian alterations in depression. The most consistent circadian abnormalities that have been described are changes in daily mood variation, brain activity, core body temperature, hormone secretion, sleep–wake cycle, motor activity, and seasonal mood variation (66). Most studies reported evidence for a blunted amplitude of the circadian rhythm (10, 67). Though, again, no grading by stress levels was examined.

Surprisingly, and somewhat opposed to our hypothesis, in those with depressive symptoms and extreme chronic stress, overall HR was lower, and decreased variability of the HR circadian rhythm was observed, but chronic stress and depressive symptoms were not associated with night-time changes in RMSSD. Regarding the findings on RMSSD, we here only analyzed night-time data and found no difference. Previously, it has been reported that patients with depression had lower RMSSD during sleep compared to those without MDD, although this relationship was partially explained by anti-depressant use (68). The here studied group did not take any anti-depressant medication, and only had depressive symptoms and effects were negligible. As the extreme stress group with depressive symptoms only comprised 10 people, the difference (or lack thereof for RMSSD) might be attributable to this small sample size. It is important to bear in mind that only very few of our healthy participants with extreme stress reported low depressive symptoms (i.e., ∼2%). The co-occurrence of high depressive symptoms and extreme chronic stress is much more common (i.e., ∼18%), be that due to direct relationship or through association with a secondary factor (such as altered cognition). The here analyzed sample of stress and low depressive symptoms is thus quite rare and albeit a small sample, it allowed us to gather preliminary data on the intriguing relationship between stress and depressive symptoms.

The allostatic load model posits that accumulated stress exposure leads to wear and tear of the body (69). Following this model, in depressive illness, there is an exhaustion of the stress system, leading to, among others, a dysregulation of the cardiovascular system (69). The latter potentially results in the observed alterations of circadian HR. It is also possible that depressive symptoms are the consequence of unresolved, chronic stress exposure (in absence of protective factors), over a sustained time-period. It has been suggested that dysregulation of the circadian system increases susceptibility to depression (70, 71). Such dysregulation might be the result of chronic stress exposure, as the circadian system interacts with stress-related neurotransmitter systems, including serotonergic neurotransmission. Therefore, a stress-induced change within the serotonin system may cause circadian dysfunction and increased vulnerability to depression (12, 72). Given that circadian misalignment is associated with decreased cardiovascular health (73), clinicians should thus be on the lookout for depressive symptomatology in patients who report high levels of stress.

It should be noted that adding hours of physical activity to our model reduced the amount of variance explained by high chronic stress. It is well known that higher physical activity is linked to resilience and better mental wellbeing (74) and inversely, that chronic stress may reduce physical activity (75), and thus these results are hardly surprising. Physical activity is also effective in reducing depressive symptoms (76). Our results may thus also point toward this direction, albeit formal analyses for moderating effects of this relationship were not conducted. Additionally, we found that participants with depressive symptoms have lower sleep quality. Sleep quality has been described as an important factor linking chronic stress and depression, potentially by moderating the effect between both (77). In their study, da Estrela et al. (77) showed that lower resting HRV was linked to poorer sleep quality. Furthermore, sleep disturbances after stress exposure have been linked to depressive symptoms (78, 79). It is a known fact that sleep is highly important for regulating physiological functions such as HR (80), and therefore it could also be that the effects we observed here are moderated through sleep quality differences. Although beyond the scope for this article, future studies could elucidate this relationship further. Lastly, in this population, the high stress groups tended to include more women than men. This has been reported before in studies using the PSS (81), as well as using other psychometric scales (82). Recently it was shown that in men, depressive symptoms are associated with lower circadian variation in vagal activity, but an opposite trend was observed in women (11). We attempted to limit sex-related biases by adding sex as a covariate to the baseline of the circadian model, but stratified analyses were not possible due to the small number of participants in the subgroups.

Of particular interest is our finding regarding stress reactivity in this healthy population. Ample research exists on HR stress reactivity in control participants [e.g., (83)], participants with depressive symptoms [see Hamilton and Alloy (57) for an overview] and in depressed patients [see Schiweck et al. (84) for an overview]. Here, depressive symptoms were associated with a blunted reactivity, but chronic stress was linked to higher reactivity. It is of course possible, that other confounding factors play a role in this relationship between blunted reactivity and depressive symptoms: next to the above-mentioned individual factors such as experience of childhood adversity (85), obesity and poor cognitive functioning (23), motivation (86), hours of sleep/sleep quality (87), and preference of “eveningness” may be associated with a blunted cardiovascular response (88). Alternatively, a true association between depressive symptoms and blunted HR reactivity is possible and has been reported in healthy populations before [e.g., (89, 90)], also for other measures: for instance, cortisol reactivity to a naturalistic stressor was abolished in those with high depressive symptoms (91).

With regard to chronic stress, literature has also identified chronic stress to influence HR reactivity to stress [e.g., (92, 93)]. In previous studies it was difficult to disentangle the effects of high chronic stress levels and depressive symptoms, since most patients with depression experience elevated stress levels, and increased stress levels in the population are also associated with an increased likelihood of depression. In light of the findings showing that blunted HR stress reactivity may be associated with motivational dysfunction, or in the case of people with cardiovascular disease even cardiovascular dysfunction (94), our study provides first data on the differential impact of both and can be used as a steppingstone for further research. In the past it has been shown that exercise therapy is highly beneficial for patients with mild to moderate depression and as supplementation even for patients with severe depression (76). Since exercise therapy targets both depressive symptoms and can prevent/improve various health conditions (cardiovascular diseases, type 2 diabetes and metabolic syndrome) (76), this form of intervention should be strongly considered for people with high levels of stress and depressive symptoms.

## Conclusion

In this study, we were able to show that high and extreme stress alone did not have any consequence on circadian rhythm, apart from a limited increase in basal HR. Yet, in the presence of depressive symptoms, extreme chronic stress levels did lead to blunted HR circadian rhythm. In addition, blunted reactivity to stressors was associated with depressive symptoms and not chronic stress. Our data suggest that using interventions which target depressive symptoms and cardiovascular health, such as exercise therapy, may be highly relevant for those with high levels of stress and depressive symptoms.

### Limitations

A number of limitations need to be mentioned for this study. As this study was cross-sectional, it does not allow to evaluate the question of temporal precedence/causality of either depressive symptoms or high stress levels. The limited sample size of people with extreme stress (a total of 20 people) also shows that this group is rather rare in the healthy population, as expected, consequently power for group comparisons was low. Furthermore, we did not assess cognition in this sample. It may very well be that altered cognition, as often encountered in depression, leads to a higher subjective experience of chronic stress. Additionally, we did not have an objective measure of chronic stress, such as e.g., hair cortisol. Moreover, the study was limited to a single physiological parameter, instead of a combination of multiple parameters. Another limitation was the lack of a clinical assessment for depressive symptoms on intake, and that other parameters which can influence HR/HRV outcomes have not been assessed. Furthermore, our model for HRV circadian analysis did not yield a sufficiently good fit, only RMSSD data during the night provides limited insight. Circadian HRV analyses would be important to draw conclusions for cardiac vagal modulation and thus yield more insight into the biology of chronic stress and depressive symptoms. As there was no psychiatric interview, the sample might include some people with undiagnosed pathology. The sample was also limited to mostly highly educated people, which may not be representative of the complete population. Other limitations related to group differences in sleep quality and sex. These variables were added as covariates to the baseline of the circadian model to reduce possible biases. Particularly sex should be investigated further in future studies since important differences in circadian rhythm abnormalities have been found.

## Data availability statement

The data analyzed in this study is subject to the following licenses/restrictions: The data that support the findings of this study are available on request. The data are not publicly available due to them containing information that could compromise research subject privacy. Requests to access these datasets should be directed to CV, chris.vanhoof@imec.be.

## Ethics statement

The studies involving human participants were reviewed and approved by the Ethics Committee Research UZ/KU Leuven. The participants provided their written informed consent to participate in this study.

## Author contributions

EL designed and performed the analyses and wrote the manuscript. CS reviewed the analyses, performed literature search, and helped with manuscript preparation. JC provided assistance during the trial and during data analysis. WD, AR, and EV provided counsel on statistical analyses and manuscript preparation. SC and CV helped with conception of the trial, counsel on statistical analyses, and manuscript preparation, as well as overall supervision. All authors contributed to the article and approved the submitted version.
