# Supplementary material for: The cumulative effect of chronic stress and depressive symptoms affects heart rate in a working population
Source: Front Psychiatry. 2022 Oct 13;13:1022298. doi: 10.3389/fpsyt.2022.1022298 (PMC9606467; doi:10.3389/fpsyt.2022.1022298)
Supplement: Supplementary file 1 [file Data_Sheet_1.docx]

Supplementary Material

# The Cumulative Effect of Chronic stress and Depressive Symptoms affects Heart Rate

# Supplementary Information 1: Details on the MIST mobile app task

The MIST mobile version was specifically designed for this experiment and is oriented at the design of Dedovic et al (2005). During the training condition, the application displayed solely the arithmetic tasks and feedback (i.e., ‘wrong’ or ‘right’) on the submitted responses. During the experimental task, a time bar, indicating a time restriction, was added, together with two performance indicators, one for the participant’s performance and one for the average performance of all participants. The latter was a false score which was kept higher than the individual performance. To encourage higher performance, participants were told they could increase their chances to win a travel voucher or dinner if they submitted the highest number of correct answers. However, the design of the task did not allow three successive correct answers, regardless of the participant’s arithmetic ability.

## Supplementary Information 2: Quality indicators for the ECG

The algorithm performs peak detection on every 10s segment and evaluates the physiological feasibility of the output against three rules. If no rule is violated, the algorithm proceeds to adaptive QRS template matching. The end-result holds a binary vector (0 = low quality,1 = high quality) with an outcome for every 10s.

**Supplementary Table 1.** *Comparison variability HR and HRV*

|  | **Variance** | | **Std. Dev.** | | **AIC** | **LogLik** |
| --- | --- | --- | --- | --- | --- | --- |
|  | *Subject* | *Day within subject* | *Subject* | *Day within subject* |  |  |
| log (HR) ~ 1\|Subject/Day | 0.012 | 0.002 | 0.109 | 0.049 | -56656.8 | 28332.4 |
| log (RMSSD) ~ 1\|Subject/Day | 0.117 | 0.016 | 0.341 | 0.128 | 30671.5 | -15331.8 |

**Supplementary Table 2.** *Result of the linear regression model on the log-transformed average HR during the night (< 6 AM).*

|  | **Estimate** | **Std. error** | **t value** | **p value** |
| --- | --- | --- | --- | --- |
| Intercept | 4.099 | 0.030 | 136.294 | **< 0.001** |
| BMI | 0.171 | 0.038 | 4.472 | **< 0.001** |
| Sex (Male) | -0.105 | 0.012 | -9.040 | **< 0.001** |
| Smoking (Yes) | 0.056 | 0.022 | 2.556 | **0.011** |
| PSS | 0.214 | 0.113 | 1.899 | 0.058 |
| PSS² | -0.177 | 0.114 | -1.549 | 0.122 |

*Abbreviations: BMI- Body Mass Index, PSS-Perceived stress scale, HR-average heart rate*

*bold, p<0.05*

*Results are presented for the final model, which was selected based on AIC, starting from the full model including all first and second order terms for DASSD and PSS.*

**Supplementary Table 3.** *Sample characteristics.* Differences are indicated for stress groups.

| **Chronic stress** | **1**  **Normal**  **(n = 390)** | | | **2**  **High**  **(n = 106)** | | | **3**  **Extreme**  **(n = 20)** | | |  | |  | |  |
| --- | --- | --- | --- | --- | --- | --- | --- | --- | --- | --- | --- | --- | --- | --- |
|  | *median* | *IQR* | *median* | | *IQR* | *median* | | *IQR* | *X²*  *value* | | *p value* | | *significant differences* | |
| Age | 39.00 | 16.00 | 38.00 | | 16.75 | 43.00 | | 10.00 | 1.94 | | 0.379 | |  | |
| BMI | 24.08 | 4.53 | 22.99 | | 5.25 | 24.48 | | 4.93 | 5.24 | | 0.073 | |  | |
| Hours of PA | 2.0 | 2.0 | 2.0 | | 2.0 | 2.0 | | 1.25 | 7.00 | | **0.030** | | 1↔2 | |
| Sex female | 41.79% |  | 61.32% | |  | 75% | |  | 19.26 | | **<0.001** | | 1$\leftrightarrow$2,3 | |
| Smoking | 7.44% |  | 6.60% | |  | 5% | |  | 0.23 | | 0.890 | |  | |
| PSQI^1^ | 4 | 3 | 5 | | 3 | 7 | | 3.75 | 42.13 | | **<0.001** | | 1$\leftrightarrow$2,3^2^ | |
| PSS | 12 | 6 | 21 | | 2.75 | 27 | | 3 | 288.89 | | **<0.001** | | 1$\leftrightarrow$2,3^3^ | |
| DASSD | 1 | 3 | 3.5 | | 4.75 | 6.5 | | 4.5 | 96.21 | | **<0.001** | | $\leftrightarrow$1$\leftrightarrow$2 $\leftrightarrow$3 | |
| HR (in bpm) | 66.91 | 10.25 | 68.37 | | 9.56 | 66.09 | | 8.94 | 6.38 | | **0.041** | | 1$\leftrightarrow$2 | |
| Std. Acc | 0.0410 | 0.0126 | 0.0404 | | 0.0132 | 0.0414 | | 0.0152 | 0.62 | | 0.735 | |  | |

*Abbreviations: BMI-Body Mass Index, PA-Physical activity, PSQI-Pittsburgh Sleep Quality Index, PSS-Perceived stress scale, DASSD-Depression scale of the Depression Anxiety Stress scale, HR-average heart rate, Std Acc- standard deviation of the magnitude of acceleration.*

*bold, p<0.05*

*^1^ The PSQI included missing values. The true number of entries were the following: n = 365 for the normative stress group, n = 97 for the high stress group and n = 18 for the extreme stress group.*

*^2^ Significance of 2↔3: p = 0.067*

*^3^ Significance of 2↔3: p = 0.083*

**Supplementary Table 4.** *Sample characteristics.* Differences are indicated for Depressive symptoms groups.

| **Depressive**  **symptoms** | **Low**  **(n = 460)** | |  | **High**  **(n = 56)** |  |  |  |
| --- | --- | --- | --- | --- | --- | --- | --- |
|  | *median* | *IQR* | | *median* | *IQR* | *W value/X²* | *p value* |
| Age | 39.00 | 16.00 | | 39.00 | 17.25 | 13045 | 0.876 |
| BMI | 23.88 | 4.68 | | 24.18 | 3.53 | 12649 | 0.827 |
| Hours of PA | 2.0 | 2.0 | | 1.5 | 2.0 | 14936 | **0.042** |
| Sex female | 46.52% |  | | 51.79% |  | 0.364 | 0.546 |
| Smoking | 6.74% |  | | 10.71% |  | 0.663 | 0.415 |
| PSQI^1^ | 4 | 3 | | 5.5 | 2.5 | 7204.5 | **<0.001** |
| PSS | 14 | 8.25 | | 21 | 7.00 | 4502 | **<0.001** |
| DASSD | 1 | 3 | | 8.5 | 3 | 0 | **<0.001** |
| HR (in bpm) | 67.37 | 10.43 | | 66.17 | 9.21 | 13090 | 0.842 |
| Std. Acc | 0.0410 | 0.0125 | | 0.0393 | 0.0138 | 14089 | 0.251 |

*Abbreviations: BMI-Body Mass Index, PA-Physical activity, PSQI-Pittsburgh Sleep Quality Index, PSS-Perceived stress scale, DASSD-Depression scale of the Depression Anxiety Stress scale, HR-average heart rate, Std Acc- standard deviation of the magnitude of acceleration.*

*bold, p<0.05*

*^1^ The PSQI included missing values. The true number of entries were the following: n = 428 for the low in depressive symptoms group and n = 52 for the high in depressive symptoms group.*

**Supplementary Table 5.** *Sample characteristics of the MIST subset.* Differences are indicated for depressive symptomatology.

| **Depressive**  **symptoms** | **Low**  **(n = 180)** | |  | **High**  **(n = 14)** |  |  |  |
| --- | --- | --- | --- | --- | --- | --- | --- |
|  | *median* | *IQR* | | *median* | *IQR* | *W value/X²* | *p value* |
| Age | 37.00 | 16.25 | | 34.50 | 12.25 | 1371.5 | 0.583 |
| BMI | 23.80 | 4.17 | | 24.66 | 3.20 | 977.5 | 0.163 |
| Hours of PA | 2.00 | 2.00 | | 1.50 | 1.00 | 1441.5 | 0.354 |
| Sex female | 51.67% |  | | 35.71% |  | 0.761 | 0.383 |
| Smoking | 7.78% |  | | 14.29% |  | 0.121 | 0.728 |
| PSS | 13 | 8.25 | | 23 | 5.75 | 477.5 | **<0.001** |
| DASSD | 1 | 3 | | 9.5 | 3.50 | 0 | **<0.001** |
| Baseline HR (in bpm) | 69.05 | 12.09 | | 73.08 | 12.67 | 928 | 0.101 |

*Abbreviations: BMI-Body Mass Index, PA-Physical activity, PSS-Perceived stress scale, DASSD-Depression scale of the Depression Anxiety Stress scale, HR-average heart rate*

*bold, p<0.05*

**Supplementary Table 6.** *Sample characteristics of the MIST subset.* Differences are indicated for chronic stress.

| **Chronic stress** | **Normal**  **(n = 152)** |  | **High**  **(n = 42)** |  |  |  |
| --- | --- | --- | --- | --- | --- | --- |
|  | *median* | *IQR* | *Median* | *IQR* | *W value/X²* | *p value* |
| Age | 37.00 | 17.25 | 37.00 | 13.00 | 3188 | 0.991 |
| BMI | 23.90 | 3.79 | 24.24 | 5.34 | 3323.5 | 0.684 |
| Hours of PA | 2.00 | 2.00 | 2.00 | 1.00 | 3553 | 0.246 |
| Sex (female) | 47.37% |  | 61.90% |  | 2.231 | 0.135 |
| Smoking (Yes) | 7.89% |  | 9.52% |  | < 0.001 | 0.981 |
| PSS | 12 | 6 | 21 | 4 | 0 | **<0.001** |
| DASSD | 1 | 3 | 4 | 4 | 1569 | **<0.001** |
| Baseline HR (in bpm) | 70.06 | 11.99 | 68.16 | 12.20 | 3295 | 0.75 |

*Abbreviations: BMI-Body Mass Index, PA-Physical activity, PSS-Perceived stress scale, DASSD-Depression scale of the Depression Anxiety Stress scale; HR-Heart rate.*

*bold, p<0.05*

**Supplementary Table 7.** *Result of the linear mixed model on circadian variations in HR.*

|  | **Estimate** | | **Std. error** | **t value** | **p value** |
| --- | --- | --- | --- | --- | --- |
| Intercept | | 4.170 | 0.012 | 336.928 | **< 0.001** |
| BMI | | 0.139 | 0.031 | 4.513 | **< 0.001** |
| Sex (Male) | | -0.073 | 0.009 | -7.825 | **< 0.001** |
| Activity index | | 1.164 | 0.017 | 67.039 | **< 0.001** |
| Smoking (Yes) | | 0.046 | 0.018 | 2.589 | **0.010** |
| Stress (High) | | 0.028 | 0.013 | 2.185 | **0.029** |
| Stress (Extreme) | | 0.006 | 0.033 | 0.170 | 0.865 |
| Depressive symptoms (High) | | 0.006 | 0.024 | 0.243 | 0.808 |
| sin (2 * pi/24 * (t)) | | -0.052 | 0.002 | -22.365 | **< 0.001** |
| cos (2 * pi/24 * (t) ) | | -0.023 | 0.002 | -9.227 | **< 0.001** |
| sin (2 * pi/12 * (t)) | | -0.023 | 0.001 | -21.051 | **< 0.001** |
| cos (2 * pi/12 * (t)) | | 0.011 | 0.001 | 9.559 | **< 0.001** |
| sin (2 * pi/8 * (t)) | | 0.002 | 0.001 | 2.509 | **0.012** |
| cos (2 * pi/8 * (t)) | | -0.001 | 0.001 | -1.578 | 0.115 |
| sin (2 * pi/6 * (t)) | | 0.010 | 0.001 | 14.125 | **< 0.001** |
| cos (2 * pi/6 * (t)) | | -0.006 | 0.001 | -8.060 | **< 0.001** |
| Stress (High) * Dep. (High) | | -0.015 | 0.034 | -0.434 | 0.664 |
| Stress (Extreme) * Dep. (High) | | -0.089 | 0.052 | -1.700 | 0.090 |
| Stress (High) * cos (2 * pi/8 * (t)) | | -0.002 | 0.002 | -1.010 | 0.313 |
| Stress (Extreme) * cos (2 * pi/8 * (t)) | | -0.006 | 0.005 | -1.108 | 0.268 |
| Dep (High) * cos (2 * pi/8 * (t)) | | -0.003 | 0.004 | -0.856 | 0.393 |
| Stress (High) * Dep. (High) * cos (2 * pi/8 * (t)) | | 0.006 | 0.005 | 1.151 | 0.250 |
| Stress (Extreme) * Dep. (High) * cos (2 * pi/8 * (t)) | | 0.018 | 0.008 | 2.184 | **0.030** |

*Abbreviations: BMI-Body Mass Index*

*bold, p<0.05*

*Results are presented for the final model predicting heart rate after log transformation, with random intercept and random slopes (AIC = -84655.2, LogLik = 42396.6 and conditional R² = 0.75). Sines and cosines terms are indicated for 24-hour cycles, 12-hour cycles, 8-hour cycles and 6-hour cycles.*

*The PSQI was added as an additional covariate to the final model but was not retained since it did not lower AIC (n = 480; PSQI included AIC = -78527.15, PSQI excluded AIC = -78528.65) and was not significant (b = 0.001, sd = 0.002, t = 0.710, p = 0.478).*

**Supplementary Table 8.** *Result of the linear mixed model on circadian variations in HR within the low depression subgroup.*

|  | **Estimate** | **Std. error** | **t value** | **p value** |
| --- | --- | --- | --- | --- |
| Intercept | 4.164 | 0.013 | 319.332 | **< 0.001** |
| BMI | 0.158 | 0.033 | 4.834 | **< 0.001** |
| Sex (Male) | -0.072 | 0.010 | -7.194 | **< 0.001** |
| Activity index | 1.163 | 0.018 | 63.496 | **< 0.001** |
| Smoking (Yes) | 0.035 | 0.019 | 1.796 | 0.073 |
| Stress (High) | 0.031 | 0.013 | 2.318 | **0.021** |
| Stress (Extreme) | 0.008 | 0.034 | 0.238 | 0.812 |
| sin (2 * pi/24 * (t)) | -0.052 | 0.002 | -20.910 | **< 0.001** |
| cos (2 * pi/24 * (t) ) | -0.023 | 0.003 | -9.001 | **< 0.001** |
| sin (2 * pi/12 * (t)) | -0.022 | 0.001 | -20.141 | **< 0.001** |
| cos (2 * pi/12 * (t)) | 0.010 | 0.001 | 8.731 | **< 0.001** |
| sin (2 * pi/8 * (t)) | 0.002 | 0.001 | 2.661 | **0.008** |
| cos (2 * pi/8 * (t)) | -0.002 | 0.001 | -2.237 | **0.026** |
| sin (2 * pi/6 * (t)) | 0.011 | 0.001 | 14.245 | **< 0.001** |
| cos (2 * pi/6 * (t)) | -0.006 | 0.001 | -7.755 | **< 0.001** |

*Abbreviations: BMI-Body Mass Index*

*bold, p<0.05*

*Results are presented for the final model predicting heart rate after log transformation, with random intercept and random slopes (AIC = -75831.6, LogLik = 37976.8, conditional R² = 0.75). Sines and cosines terms are indicated for 24-hour cycles, 12-hour cycles, 8-hour cycles and 6-hour cycles.*

**Supplementary Table 9.** *Result of the linear mixed model on circadian variations in HR within the high depression subgroup.*

|  | **Estimate** | **Std. error** | **t value** | **p value** |
| --- | --- | --- | --- | --- |
| Intercept | 4.228 | 0.026 | 165.437 | **< 0.001** |
| Sex (Male) | -0.087 | 0.024 | -3.613 | **< 0.001** |
| Activity index | 1.191 | 0.055 | 21.702 | **< 0.001** |
| Smoking (Yes) | 0.084 | 0.038 | 2.236 | **0.025** |
| Stress (High) | 0.005 | 0.027 | 0.196 | 0.845 |
| Stress (Extreme) | -0.084 | 0.035 | -2.408 | **0.016** |
| sin (2 * pi/24 * (t)) | -0.056 | 0.007 | -7.952 | **< 0.001** |
| cos (2 * pi/24 * (t) ) | -0.020 | 0.008 | -2.324 | **0.020** |
| sin (2 * pi/12 * (t)) | -0.033 | 0.006 | -5.521 | **< 0.001** |
| cos (2 * pi/12 * (t)) | 0.013 | 0.003 | 4.159 | **< 0.001** |
| sin (2 * pi/8 * (t)) | 0.000 | 0.002 | 0.061 | 0.952 |
| cos (2 * pi/8 * (t)) | -0.005 | 0.003 | -1.711 | 0.087 |
| sin (2 * pi/6 * (t)) | 0.008 | 0.002 | 4.255 | **< 0.001** |
| cos (2 * pi/6 * (t)) | -0.005 | 0.002 | -2.741 | **0.006** |
| Stress (High) * sin (2 * pi/12 * (t)) | 0.008 | 0.008 | 1.000 | 0.317 |
| Stress (Extreme) * sin (2 * pi/12 * (t)) | 0.022 | 0.010 | 2.176 | **0.030** |
| Stress (High) * cos (2 * pi/8 * (t)) | 0.004 | 0.004 | 1.037 | 0.300 |
| Stress (Extreme) * cos (2 * pi/8 * (t)) | 0.011 | 0.005 | 2.136 | **0.033** |

*bold, p<0.05*

*Results are presented for the final model predicting heart rate after log transformation, with random intercept and random slopes (AIC = -8740.7, LogLik = 4410.3 and conditional R² = 0.72). Sines and cosines terms are indicated for 24-hour cycles, 12-hour cycles, 8-hour cycles and 6-hour cycles. During model selection, the random slopes for cos ( (*$\frac{2\pi}{8}$*h), sin ( (*$\frac{2\pi}{6}$*h) and cos ( (*$\frac{2\pi}{6}$*h) were dropped.*

**Supplementary Table 10.** *Result of the linear mixed model on circadian variations in HR – including the hours of physical activity (PA).*

|  | **Estimate** | | **Std. error** | **t value** | **p value** |
| --- | --- | --- | --- | --- | --- |
| Intercept | | 4.209 | 0.014 | 299.427 | **< 0.001** |
| BMI | | 0.120 | 0.030 | 3.975 | **< 0.001** |
| Sex (Male) | | -0.062 | 0.009 | -6.645 | **< 0.001** |
| Hours of PA | | -0.082 | 0.015 | -5.449 | **< 0.001** |
| Activity index | | 1.164 | 0.017 | 67.045 | **< 0.001** |
| Smoking (Yes) | | 0.039 | 0.017 | 2.237 | **0.026** |
| Stress (High) | | 0.022 | 0.013 | 1.749 | 0.081 |
| Stress (Extreme) | | 0.001 | 0.032 | 0.026 | 0.979 |
| Dep (High) | | -0.009 | 0.024 | -0.366 | 0.714 |
| sin (2 * pi/24 * (t)) | | -0.052 | 0.002 | -22.364 | **< 0.001** |
| cos (2 * pi/24 * (t) ) | | -0.023 | 0.002 | -9.226 | **< 0.001** |
| sin (2 * pi/12 * (t)) | | -0.023 | 0.001 | -21.051 | **< 0.001** |
| cos (2 * pi/12 * (t)) | | 0.011 | 0.001 | 9.559 | **< 0.001** |
| sin (2 * pi/8 * (t)) | | 0.002 | 0.001 | 2.505 | **0.013** |
| cos (2 * pi/8 * (t)) | | -0.001 | 0.001 | -1.572 | 0.117 |
| sin (2 * pi/6 * (t)) | | 0.010 | 0.001 | 14.129 | **< 0.001** |
| cos (2 * pi/6 * (t)) | | -0.006 | 0.001 | -8.059 | **< 0.001** |
| Stress (High) * Dep (High) | | -0.001 | 0.033 | -0.026 | 0.979 |
| Stress (Extreme) * Dep (High) | | -0.065 | 0.051 | -1.271 | 0.204 |
| Stress (High) * cos (2 * pi/8 * (t)) | | -0.002 | 0.002 | -1.014 | 0.311 |
| Stress (Extreme) * cos (2 * pi/8 * (t)) | | -0.006 | 0.005 | -1.112 | 0.266 |
| Dep (High) * cos (2 * pi/8 * (t)) | | -0.003 | 0.004 | -0.859 | 0.391 |
| Stress (High) * Dep (High) * cos (2 * pi/8 * (t)) | | 0.006 | 0.005 | 1.153 | 0.249 |
| Stress (Extreme) * Dep (High) * cos (2 * pi/8 * (t)) | | 0.018 | 0.008 | 2.187 | **0.029** |

*Abbreviations: BMI-Body Mass Index, PA-Physical activity*

*bold, p<0.05*

*Results are presented for the final model predicting heart rate after log transformation, with random intercept and random slopes (AIC = -84681.4, logLik = 42410.7 and R² = 0.75). Sines and cosines terms are indicated for 24-hour cycles, 12-hour cycles, 8-hour cycles and 6-hour cycles.*

**Supplementary Table 11.** *Result of the linear mixed model on circadian variations in HR within the low depression subgroup – including the hours of physical activity (PA).*

|  | **Estimate** | **Std. error** | **t value** | **p value** |
| --- | --- | --- | --- | --- |
| Intercept | 4.215 | 0.014 | 299.010 | **< 0.001** |
| BMI | 0.132 | 0.032 | 4.110 | **< 0.001** |
| Sex (Male) | -0.063 | 0.010 | -6.414 | **< 0.001** |
| Hours of PA | -0.087 | 0.016 | -5.443 | **< 0.001** |
| Activity index | 1.163 | 0.018 | 63.501 | **< 0.001** |
| sin (2 * pi/24 * (t)) | -0.052 | 0.002 | -20.911 | **< 0.001** |
| cos (2 * pi/24 * (t) ) | -0.023 | 0.003 | -9.000 | **< 0.001** |
| sin (2 * pi/12 * (t)) | -0.022 | 0.001 | -20.140 | **< 0.001** |
| cos (2 * pi/12 * (t)) | 0.010 | 0.001 | 8.733 | **< 0.001** |
| sin (2 * pi/8 * (t)) | 0.002 | 0.001 | 2.653 | **0.008** |
| cos (2 * pi/8 * (t)) | -0.002 | 0.001 | -2.231 | **0.026** |
| sin (2 * pi/6 * (t)) | 0.011 | 0.001 | 14.251 | **< 0.001** |
| cos (2 * pi/6 * (t)) | -0.006 | 0.001 | -7.755 | **< 0.001** |

*Abbreviations: BMI-Body Mass Index, PA-Physical activity*

*bold, p<0.05*

*Results are presented for the final model predicting heart rate after log transformation, with random intercept and random slopes (AIC = -75855.3, logLik = 37986.7 and R² = 0.75). Sines and cosines terms are indicated for 24-hour cycles, 12-hour cycles, 8-hour cycles and 6-hour cycles. Following the AIC-based model selection, the effect of chronic stress was dropped from the group model of the low depression subgroup.*

**Supplementary Table 12.** *Result of the linear mixed model on circadian variations in HR within the high depression subgroup – including the hours of physical activity (PA).*

|  | **Estimate** | **Std. error** | **t value** | **p value** |
| --- | --- | --- | --- | --- |
| Intercept | 4.236 | 0.026 | 165.415 | **< 0.001** |
| Sex (Male) | -0.064 | 0.024 | -2.624 | **0.009** |
| Hours of PA | 1.191 | 0.055 | 21.709 | **< 0.001** |
| Activity index | -0.080 | 0.038 | -2.109 | **0.035** |
| Smoking (Yes) | 0.089 | 0.036 | 2.446 | **0.014** |
| Stress (High) | 0.016 | 0.026 | 0.636 | 0.525 |
| Stress (Extreme) | -0.061 | 0.035 | -1.759 | 0.079 |
| sin (2 * pi/24 * (t)) | -0.056 | 0.007 | -7.952 | **<0.001** |
| cos (2 * pi/24 * (t) ) | -0.020 | 0.008 | -2.323 | **0.020** |
| sin (2 * pi/12 * (t)) | -0.033 | 0.006 | -5.517 | **<0.001** |
| cos (2 * pi/12 * (t)) | 0.013 | 0.003 | 4.161 | **<0.001** |
| sin (2 * pi/8 * (t)) | 0.000 | 0.002 | 0.059 | 0.953 |
| cos (2 * pi/8 * (t)) | -0.005 | 0.003 | -1.714 | 0.087 |
| sin (2 * pi/6 * (t)) | 0.008 | 0.002 | 4.253 | **<0.001** |
| cos (2 * pi/6 * (t)) | -0.005 | 0.002 | -2.743 | **0.006** |
| Stress (High) * sin (2 * pi/12 * (t)) | 0.008 | 0.008 | 0.998 | 0.318 |
| Stress (Extreme) * sin (2 * pi/12 * (t)) | 0.022 | 0.010 | 2.176 | **0.030** |
| Stress (High) * cos (2 * pi/8 * (t)) | 0.004 | 0.004 | 1.038 | 0.299 |
| Stress (Extreme) * cos (2 * pi/8 * (t)) | 0.011 | 0.005 | 2.141 | **0.032** |

*Abbreviations: PA-Physical activity*

*bold, p<0.05*

*Results are presented for the final model predicting heart rate after log transformation, with random intercept and random slopes (AIC = -8742.5, logLik = 4412.2, and R² = 0.71). Sines and cosines terms are indicated for 24-hour cycles, 12-hour cycles, 8-hour cycles and 6-hour cycles. During model selection, the random slopes for cos ( (*$\frac{2\pi}{8}$*h), sin ( (*$\frac{2\pi}{6}$*h) and cos ( (*$\frac{2\pi}{6}$*h) were dropped.*

**Supplementary Table 13.** *Result of the linear mixed model on night data in RMSSD.*

|  | **Estimate** | **Std. error** | **t value** | **p value** |
| --- | --- | --- | --- | --- |
| Intercept | **4.201** | **0.055** | **76.455** | **<0.001** |
| Age | **-0.701** | **0.068** | **-10.313** | **<0.001** |
| BMI | **-0.304** | **0.109** | **-2.785** | **0.006** |
| Sex (Male) | -0.041 | 0.033 | -1.249 | 0.212 |
| Hours of PA | 0.060 | 0.053 | 1.129 | 0.259 |
| Activity index | **1.724** | **0.145** | **11.860** | **<0.001** |
| Smoking | -0.097 | 0.061 | -1.589 | 0.113 |
| Hour | **0.016** | **0.002** | **6.652** | **<0.001** |
| Stress (High) | 0.041 | 0.047 | 0.863 | 0.389 |
| Stress (Extreme) | 0.177 | 0.120 | 1.474 | 0.141 |
| Dep (High) | 0.088 | 0.088 | 1.000 | 0.318 |
| Stress (High) * Dep (High) | -0.099 | 0.122 | -0.814 | 0.416 |
| Stress (Extreme) * Dep (High) | -0.210 | 0.189 | -1.109 | 0.268 |
| Hour * Stress (High) | -0.007 | 0.006 | -1.325 | 0.186 |
| Hour * Stress (Extreme) | 0.000 | 0.014 | -0.031 | 0.976 |
| Hour * Dep (High) | -0.007 | 0.011 | -0.678 | 0.498 |
| Hour * Stress (High) * Dep (High) | 0.002 | 0.015 | 0.149 | 0.882 |
| Hour * Stress (Extreme) * Dep (High) | -0.007 | 0.023 | -0.321 | 0.749 |

*Abbreviations: BMI-Body Mass Index, PA-Physical activity*

*bold, p<0.05*

*Results are presented for the full model predicting heart rate after log transformation, with random intercept and random slope for Hour (AIC = 9796.1, LogLik = -4876.0 and conditional R² = 0.63)*

**Supplementary Table 14.** *Mean differences in heart rate between MIST conditions detailed per depression subgroup.* Differences are indicated in a pairwise manner.

| **Depressive**  **symptoms** | **Low**  **(n = 180)** | | **High**  **(n = 14)** | |
| --- | --- | --- | --- | --- |
|  | **Mean difference** | **p-value** | **Mean difference** | **p- value** |
| Baseline – Training | -0.008 | 0.101 | -0.012 | 0.689 |
| Baseline – Stress | -0.032 | **< 0.001** | -0.008 | 0.728 |
| Baseline – Recovery | -0.002 | 0.736 | 0.015 | 0.689 |
| Training – Stress | -0.024 | **< 0.001** | 0.004 | 0.821 |
| Training – Recovery | 0.007 | 0.162 | 0.027 | 0.470 |
| Stress – Recovery | 0.031 | **< 0.001** | 0.023 | 0.470 |

*bold, p<0.05*

**Supplementary Table 15.** *Descriptive data and changes in heart rate between training and stress for all participants with depressive symptomatology in the MIST dataset.*

| **Participant** | **Sex** | **BMI** | **Hour of sports** | **PSS** | **DASSD** | **Baseline HR (BPM)** | **Change score** |
| --- | --- | --- | --- | --- | --- | --- | --- |
| **1** | M | 25-30 | 3 | 23 | 12 | 60.06 | -6.93 |
| **2** | F | 25-30 | 1 | 25 | 11 | 71.79 | -5.80 |
| **3** | F | 30-35 | 1 | 15 | 7 | 75.20 | -2.61 |
| **4** | M | 25-30 | 1 | 19 | 8 | 79.95 | -2.21 |
| **5** | M | 20-25 | 2 | 11 | 7 | 60.10 | -1.96 |
| **6** | M | 20-25 | 4 | 24 | 13 | 82.49 | -1.94 |
| **7** | M | 25-30 | 0 | 27 | 11 | 94.02 | -0.92 |
| **8** | M | 20-25 | 0 | 7 | 7 | 71.25 | -0.81 |
| **9** | M | 20-25 | 0 | 18 | 7 | 83.74 | -0.14 |
| **10** | F | 20-25 | 2 | 23 | 10 | 73.98 | 0.10 |
| **11** | F | 18-20 | 1 | 24 | 10 | 68.49 | 2.74 |
| **12** | F | 20-25 | 2 | 24 | 10 | 72.18 | 2.99 |
| **13** | M | 20-25 | 2 | 23 | 9 | 87.82 | 4.65 |
| **14** | M | 20-25 | 3 | 20 | 9 | 62.82 | 8.96 |


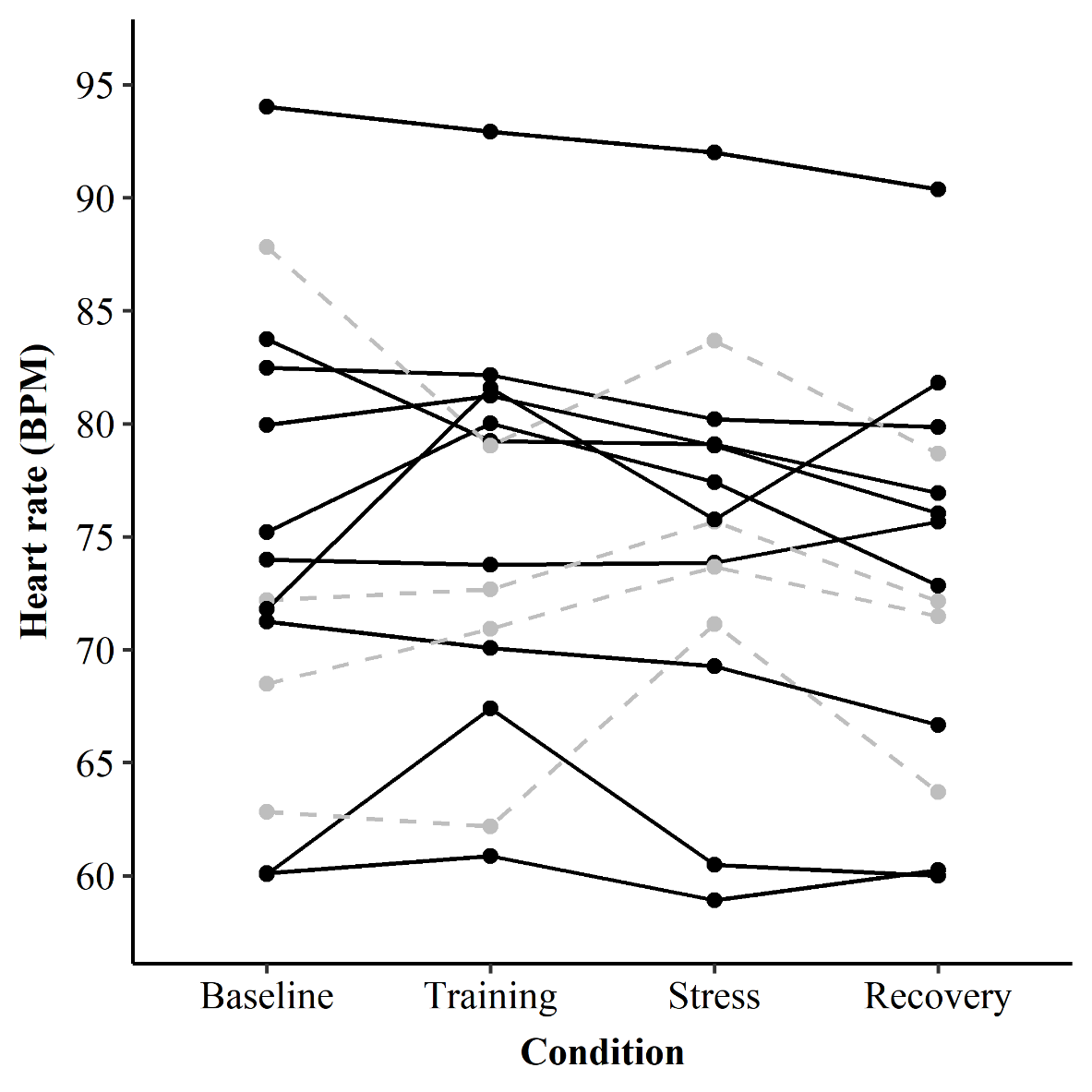


**Figure S1.** *Heart rate averaged per condition of the MIST for all participants with depressive symptomatology (n = 14).* Black solid lines indicate a negative or close to zero change from training to stress; grey dotted lines indicate a positive change.

**Supplementary Table 16.** *Mean differences in heart rate between MIST conditions detailed per stress subgroup.* Differences are indicated in a pairwise manner.

| **Chronic stress** | **Normative stress**  **(n = 152)** | | **High stress**  **(n = 42)** | |
| --- | --- | --- | --- | --- |
|  | **Mean difference** | **p-value** | **Mean difference** | **p- value** |
| Baseline – Training | -0.006 | 0.269 | -0.018 | 0.0842 |
| Baseline – Stress | -0.027 | **< 0.001** | -0.044 | **<0.001** |
| Baseline – Recovery | 0.0002 | 0.973 | -0.002 | 0.808 |
| Training – Stress | -0.021 | **< 0.001** | -0.026 | **0.011** |
| Training – Recovery | 0.006 | 0.269 | 0.016 | 0.116 |
| Stress – Recovery | 0.027 | **< 0.001** | 0.041 | **<0.001** |

*bold, p<0.05*
